# DOCUMENTATION OF LIFE-SUSTAINING TREATMENT PREFERENCES IN A NATIONAL COHORT OF VETERAN DECEDENTS

**DOI:** 10.1093/geroni/igae098.1895

**Published:** 2024-12-31

**Authors:** Lena Makaroun, Yaming Li, Maria Mor, Stephen Morris, Ann-Marie Rosland, Tony Rosen, Ann Kutney-Lee, Carolyn Thorpe

**Affiliations:** VA Pittsburgh Healthcare System, Pittsburgh, Pennsylvania, United States; VA Pittsburgh Healthcare System, Pittsburgh, Pennsylvania, United States; VA Pittsburgh Healthcare System, Pittsburgh, Pennsylvania, United States; VA Pittsburgh Healthcare System, Pittsburgh, Pennsylvania, United States; VA Pittsburgh Healthcare System, Pittsburgh, Pennsylvania, United States; Weill Cornell Medicine, New York, New York, United States; Corporal Michael J. Crescenz VA Medical Center, Philadelphia, Pennsylvania, United States; University of North Carolina, Chapel Hill, North Carolina, United States

## Abstract

In 2018, the Veterans Health Administration (VHA) introduced the Life-Sustaining Treatment (LST) Decisions Initiative to standardize documentation of Veterans’ goals and preferences in the electronic health record (EHR). The objective of this retrospective cohort study was to assess LST completion patterns and sociodemographic, clinical, and care utilization factors associated with completion in a cohort of 23,591 VHA-engaged Veterans aged ≥65 years who died in 2021. We used VHA EHR and Medicare claims data and multivariable logistic regression to examine factors associated with LST completion. Of the cohort, 6,684 (28%) completed an LST. LST documentation occurred a median of 142 days before death; 2,260 (34%) were completed by a surrogate. LST completion was markedly higher in those with an inpatient or VA nursing home (CLC) stay in the 2-years before death, so we estimated models stratified by the presence (n = 7,761) or absence (n = 15,830) of any inpatient/CLC stay. In both strata, older age, higher comorbidity, and frailty were associated with LST completion (p < 0.001). Among Veterans without an inpatient/CLC stay, being unmarried, having a child as next of kin, higher levels of VA financial benefits, and dementia diagnosis were also associated (p < 0.001). Race/ethnicity and gender were not associated with LST completion. LST completion before death is strongly tied to utilization of VHA acute and nursing home care, and generally occurs close to the time of death and often by a surrogate. Opportunities may exist to improve documentation of patient preferences in outpatient settings farther in advance of health declines.

